# Dedicated biomass crops can enhance biodiversity in the arable landscape

**DOI:** 10.1111/gcbb.12312

**Published:** 2015-11-30

**Authors:** Alison J. Haughton, David A. Bohan, Suzanne J. Clark, Mark D. Mallott, Victoria Mallott, Rufus Sage, Angela Karp

**Affiliations:** ^1^ Rothamsted ResearchWest CommonHarpendenHertfordshireAL5 2JQUK; ^2^ INRA, UMR 1347 Agroécologie, Pôle ECOLDUR17 rue SullyDijon CEDEX21065France; ^3^ 168 Putteridge RoadLutonLU2 8HJUK; ^4^ Game and Wildlife Conservation TrustBurgate ManorFordingbridgeHampshireSP6 1EFUK

**Keywords:** biodiversity indicators, bioenergy, biomass crops, canonical variates analysis, functional traits, invertebrates, miscanthus, seedbank, short rotation coppiced willow, weed biomass

## Abstract

Suggestions that novel, non‐food, dedicated biomass crops used to produce bioenergy may provide opportunities to diversify and reinstate biodiversity in intensively managed farmland have not yet been fully tested at the landscape scale. Using two of the largest, currently available landscape‐scale biodiversity data sets from arable and biomass bioenergy crops, we take a taxonomic and functional trait approach to quantify and contrast the consequences for biodiversity indicators of adopting dedicated biomass crops on land previously cultivated under annual, rotational arable cropping. The abundance and community compositions of biodiversity indicators in fields of break and cereal crops changed when planted with the dedicated biomass crops, miscanthus and short rotation coppiced (SRC) willow. Weed biomass was consistently greater in the two dedicated biomass crops than in cereals, and invertebrate abundance was similarly consistently higher than in break crops. Using canonical variates analysis, we identified distinct plant and invertebrate taxa and trait‐based communities in miscanthus and SRC willows, whereas break and cereal crops tended to form a single, composite community. Seedbanks were shown to reflect the longer term effects of crop management. Our study suggests that miscanthus and SRC willows, and the management associated with perennial cropping, would support significant amounts of biodiversity when compared with annual arable crops. We recommend the strategic planting of these perennial, dedicated biomass crops in arable farmland to increase landscape heterogeneity and enhance ecosystem function, and simultaneously work towards striking a balance between energy and food security.

## Introduction

Anthropogenic‐induced climate change continues to be the single, overriding challenge to the future of humans and ecosystems, and reductions in emissions of CO_2_ are essential to limit the risks of climate change (IPCC, 2014). Balancing the food and fuel security demands of a growing human population, in the context of climate change, has led to a global drive to increase production from land that has resulted in unforeseen land use conflicts, particularly for crops traditionally grown for food being diverted for use in the transport biofuel industry (Searchinger *et al*., 2015). These conflicts compound genuine concerns that a shift in focus on to cheaper sources of gas, including the recent developments in the shale gas industry, could disrupt progress in the development and adoption of sustainable renewable technologies and significantly delay efforts to further reduce global emissions of CO_2_ (Davis & Shearer, 2014; Jackson *et al*., 2014; McJeon *et al*., 2014).

Non‐food, perennial, dedicated biomass crops, such as trees grown as short rotation coppice and grasses, are potentially integral to reducing CO_2_ emissions and many studies have documented positive benefits of growing perennial biomass crops, including for ecosystem services (Berndes *et al*., 2008; Baum *et al*., 2013a; Meehan *et al*., 2013) and biodiversity (Haughton *et al*., 2009; Rowe *et al*., 2009; Dauber *et al*., 2010; Baum *et al*., 2012; Stanley & Stout, 2013; Bourke *et al*., 2014). However, much of the ecological evidence is directly (e.g. Rowe *et al*., 2009) or indirectly (e.g. Holland *et al*., 2015) based on studies conducted on small, temporal (single samples within a single season), spatial (localized, experimental plots) scales, whilst sustainability concerns relate to longer term, landscape‐scale expansion (Fargione, 2010; Dauber & Bolte, 2014). Furthermore, many studies assess biodiversity taxa of one type of biomass crop, without drawing comparisons with the land uses they may replace (see review by Dauber *et al*., 2010) and use coarse levels of identification (e.g. Rowe *et al*., 2011) resulting in misleading interpretation of responses for ecosystem service provision (e.g. Holland *et al*., 2015). Nevertheless, models using data derived from small‐scale experiments have predicted that perennial, dedicated biomass crops could have beneficial environmental impacts if integrated into agricultural landscapes (e.g. (Meehan *et al*., 2010; Tilman *et al*., 2009). There have been well documented declines in farmland biodiversity and ecosystem service provision in the latter half of the 20th Century (e.g. Donald *et al*., 2001; Bianchi *et al*., 2006; Storkey *et al*., 2012; Woodcock *et al*., 2014; Senapathi *et al*., 2015). These reductions in biodiversity and ecosystem function have been attributed to an homogenization of the farmed landscape, in terms of reduction in the area and diversity of semi‐natural habitats and diversity of on‐farm cropping and management systems (Benton *et al*., 2003; Bianchi *et al*., 2006). It is therefore important to test whether cultivating perennial, dedicated biomass crops in annual arable crop‐dominated landscapes could be used to enhance and conserve farmland biodiversity and ecosystem function.

The agronomic management and growth characteristics of perennial, dedicated biomass crops, such as willows (*Salix* spp), poplars (*Populus* spp) and miscanthus (*Miscanthus* spp.), contrast with those of food crops typically grown for biofuel (e.g. wheat, maize, soy). Once established, these crops can reach 3–4 m in height and have the potential to produce large yields from very low fertilizer and pesticide inputs and provide structure in the landscape right through the winter, as they are normally harvested after senescence (miscanthus) and leaf drop (usually between December and April). As they are perennials, remaining *in situ* for ca. 20 years, the soil is not cultivated annually and they provide more stable habitats punctuated only by annual (for energy grasses) or triennial (for trees grown as short rotation coppice) harvesting. For trees like poplar, that are also grown as short rotation forestry, harvesting cycles are even longer (>15 years).

Planting perennial biomass crops in farmland should, therefore, result in contrasting abundances and compositions of plants and invertebrates compared with annual arable crops, reflecting differences in both crop growth and management. To test this, we carried out extensive sampling of established fields of two perennial, dedicated biomass crops [miscanthus and short rotation coppiced (SRC) willows] and used taxonomic, functional trait and phylogenetic groupings to compare the abundance and community compositions of key biodiversity indicators with those of arable crops. Thus, we test the null hypothesis that there is no change in biodiversity in perennial, dedicated biomass crops planted on land previously under annual arable crop management.

## Materials and methods

### Experimental design

We undertook the most intensive temporal‐ and spatial‐scale sampling of biodiversity in perennial biomass crops reported to date (Karp *et al*., 2009) and compared these data with the most complete study of biodiversity previously carried out in arable crops (Coghlan, 2003; Perry *et al*., 2003) that is currently available. Although these large‐scale experiments were carried out independently of each other in different years, they were designed using the same methodologies, such that indicators of weed and invertebrate biodiversity were intensively sampled across entire, commercial fields over a single growing season and represent the most comprehensive, standardized assessment of regional‐ and national‐scale patterns of biodiversity in the farmed landscape of Great Britain. One concern with comparing data collected at different times is that populations of biodiversity indicators in farmland, per se, may have changed, making such a comparison problematic. Butterfly Lepidoptera are used as an indicator of environmental change (Defra, 2014), in part because they exhibit rapid (between‐year) response to environmental stresses. Butterfly populations were found to be stable during the period 2000–2006, when these studies were carried out (Defra, 2014) and have been used previously (Haughton *et al*., 2009) to provide confidence that any differences in abundance and community composition in the crop types are crop management‐mediated.

### Biomass crops

Using questionnaires similar to those used for study site selection in the Farm Scale Evaluation (FSE) (Champion *et al*., 2003), we selected 17 established fields of miscanthus and 15 of SRC willows that were distributed in the East Midlands, South‐west and Southern regions of England and reflected the geographical locations of commercial dedicated biomass crops (Table S1). All study fields were in standard commercial production on land that had previously been used for arable crop production and represented a range of inherent weediness from farms of varying cropping intensities that yielded between 7 and 11 tonnes winter wheat ha^−1^. The fields were planted between 1999 and 2004 and the fields of miscanthus had been harvested annually in the winter, while the SRC willows had passed through at least two coppice rotations and were due to be harvested during the winter following data collection. The biomass crops were thus representative of established, and for SRC willows, mature phase crops.

### Arable crops

The FSEs of genetically modified, herbicide‐tolerant break crops (Firbank *et al*., 2003) have previously been used to compare butterfly abundance in field margins of arable and dedicated biomass crops (Haughton *et al*., 2009). Thus, the data for the arable crops came from 255 fields sampled as part of the FSEs (Champion *et al*., 2003; Firbank *et al*., 2003; Bohan *et al*., 2005), made up of 65 fields of spring‐sown beet (*Beta vulgaris* L.), 58 fields of spring‐sown maize (*Zea mays* L.), 67 fields of spring‐sown oilseed rape (*Brassica napus* L.) and 65 fields of winter‐sown oilseed rape (*B. napus* L.). The fields represented the range of agricultural and environmental conditions found in commercial practice with regard to geographical distribution, agronomy, soil type and field size (Champion *et al*., 2003; Bohan *et al*., 2005) and treatment effects on the abundance of plant and invertebrate indicators were shown not to co‐vary with year, study site or geographical location (Haughton *et al*., 2003; Heard *et al*., 2003; Bohan *et al*., 2005). The FSEs used a split‐field design, where the effect on biodiversity indicators of ‘conventional’ arable practice was compared with that of a modified herbicide management regime associated with genetically modified, herbicide‐tolerant break crops. Here, only data from the conventional half of the split field (herein after termed ‘field’) are used. The crops were established from 2000 to 2002 and sampled throughout the growing seasons from 2000 to 2003. In the years subsequent to growing contrasting GMHT and conventional varieties of break crops, farmers followed their usual rotation and the fields were sown with a non‐GMHT crop and plant biodiversity indicators were assessed for the first 2 years following the FSEs. Biodiversity data for the cereal crops came from these follow‐up assessments of the conventional half of the split field (Heard *et al*., 2005) in fields of inter‐sown barley (*n* = 19) and winter‐sown wheat (*n* = 72).

### Weeds

Methods for sampling biodiversity indicators were standardized for all crops, using the approach taken in the FSEs, described in detail in (Firbank *et al*., 2003; Haughton *et al*., 2003; Heard *et al*., 2003; Bohan *et al*., 2005). A total of twelve, evenly spaced transects, extending 32 m into the crops, were placed around and perpendicular to the field edges (Firbank *et al*., 2003) and biodiversity indicators were sampled as follows. Soil core samples of the seedbank were taken from five loci at 2 and 32 m along four transects, prior to the break crops being sown (year *t*), and at 1 (*t* + 1) and 2 (*t* + 2) years after drilling, and in April in the biomass crops. The seeds contained within the cores were germinated and identified following the methods in Heard *et al*. (2003). Abundance is reported here as the density (numbers m^−2^) to a depth of 0.15 m, where one seed per field sample was equivalent to 18.75 m^−2^ (Heard *et al*., 2005). Seedbank data representing the effect of the four main conventional break crops of the FSE were taken a year after drilling, year *t* + 1 (Heard *et al*., 2003) and data that reflect the effect of growing cereals in the year subsequent to the break crops were taken from year *t* + 2 samples of those fields sown to cereals in year *t* + 1 (Heard *et al*., 2005).

Noncrop plant (weed) biomass, representative of a single crop growing season, was sampled in 1 m × 1 m quadrats at 2 and 32 m along all 12 transects in the month before harvest for the arable crops, and in August for the biomass crops. All plants rooted within the quadrat were cut at ground level, identified and sorted into species and dried for 24 h at 80 °C before weighing (Heard *et al*., 2003). Biomass data reported here are g m^−2^. Plant species were assigned to monocot or eudicot phylogenetic group (APG, 2003) following (Stace, 2010) and allocated to primary growth strategy following (Grime *et al*., 2007) prior to analysis (see Table S2).

### Invertebrates

Within‐crop invertebrates from the soil and weeds were sampled using a Vortis suction sampler (Arnold 1994), where five, 10‐second ‘sucks’ were taken 1 m apart at 2 and 32 m along three transects in June (Haughton *et al*., 2003) and identified to various taxonomic levels and assigned to appropriate trophic (functional) group for analysis (Table 1) (Hawes *et al*., 2003). An area of 0.6 m^2^ per field was sampled and abundance is reported here as density of invertebrates m^−2^.

**Table 1:** Levels of identification and assignment to trophic group of invertebrates

Taxa	Level of identification	Trophic group			
		Detritivore	Herbivore	Predator	Mix
Collembola	Family	y			
Orthoptera	Order		y		
Hemiptera					
Heteroptera	Species		y	y	y
Auchenorrhyncha	Sub‐order		y		
Aphidoidea	Superfamily		y		
Neuroptera	Order			y	
Lepidoptera					
Larvae	Order		y		
Diptera	Order				y
Hymenoptera					
Symphyta larvae	Sub‐order		y		
Parasitica	Superfamily			y	
Coleoptera					
Coccinellidae	Family	y		y	
Curculionidae	Family		y		
Staphylinidae	Family				y
Carabidae	Species		y	y	
Others	Order				y
Araneae					
Linyphiidae	Family			y	
*Tenuiphantes tenuis*	Species			y	
*Erigone*	Genus			y	
*Oedothorax*	Genus			y	
Others	Order			y	

### Statistical analyses

To determine whether the densities of phylogenetic and growth strategy groups of weeds and trophic groups of invertebrates differed between biomass and arable crops, field totals were transformed to common logarithms, after adding an offset of one to seedbank and invertebrate counts and 0.005 to biomass measurements. Sites for which the total count was zero or one were excluded (c.f. Heard *et al*., 2003). The number of fields included in each analysis is reported as *N*. For each biodiversity indicator group and biomass‐arable crop comparison of interest, the null hypothesis of no difference between means (H_0_: *δ* = 0, H_1_: *δ* ≠ 0, where $\hat{\delta }$ = *d*) was tested using a *t*‐test, with degrees of freedom adjusted using Satterthwaite's formula when crop variances were unequal (based on an *F*‐test, *P* > 0.05). Crop means are presented back‐transformed to the original scales. Relative crop effects for each biodiversity indicator group are reported as *R*, the multiplicative ratio (biomass crops : arable crops), calculated as *R* = 10^*d*^, where *d* is the difference between the crop means on the logarithmic scale. Upper and lower 95% confidence limits for *δ* were back‐transformed similarly to give confidence limits for the true value of *R*.

Canonical variates analysis (CVA) (Gardner *et al*., 2006) was used to detect differences in the weed and invertebrate communities of miscanthus, SRC willows and break crops; and for weeds only, between miscanthus, SRC willows and cereal crops. To avoid the effects of dominance of a few, highly abundant species (Smith *et al*., 2008), field abundance of individual weed and invertebrate taxa or grouping was transformed to proportions of the total abundance per field. Significant differences reported for the compositions of weed and invertebrate communities therefore indicate differences in proportions rather than abundance. Taxa that were considered to have occurred by chance were excluded from the analysis, such that data for taxa that were present either in only a single crop with an abundance of <1%, or in two or more crops at <0.1% abundance were removed. Where removal of these low‐abundance taxa resulted in the remaining proportion of abundance at individual sites being less than 80% of the original site total, these sites were removed from the analysis. Proportion data were arcsine square‐root transformed (Sokal & Rohlf, 2012) prior to analysis, with crop type (miscanthus, SRC willows, break, cereal crops) as the grouping factor. All analyses were done using GenStat 17th Edition (VSNI, 2014).

## Results

### Seedbank

**Figure 1:**
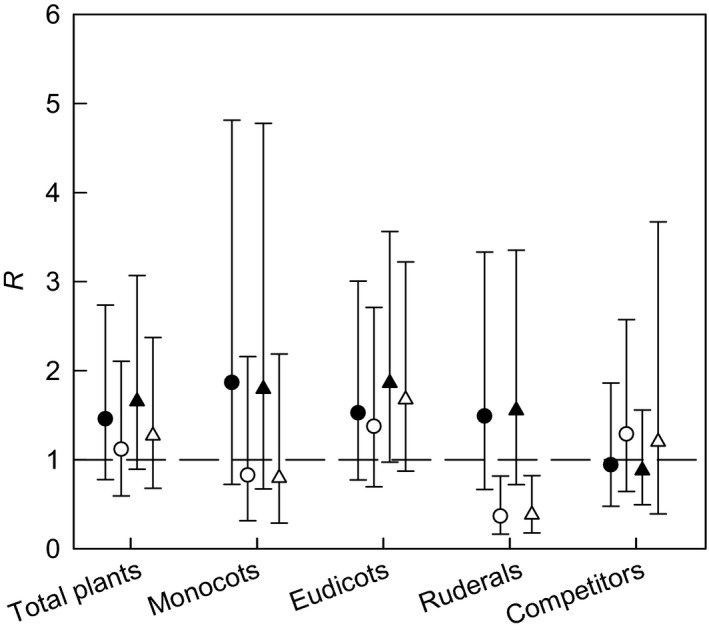
Ratio (*R*) of seed density in the seedbanks of miscanthus and SRC willows to break and cereal crops. Solid symbols: miscanthus; open symbols: SRC willows; circles: break crops; triangles: cereals. *R* is computed as 10^*d*^, where *d* is the difference between the means (over sites) of the logarithmically transformed seed density m^−2^ per field to a depth of 0.15 m. Dashed line is line of equality (*d* = 0 or *R* = 1). Error bars are 95% confidence limits for *R*, also back‐transformed to the ratio scale (hence asymmetry).

Seedbank densities tended not to differ between biomass and arable crops for all plant groupings (Fig. 1, Table 2). Total seedbank densities did not differ in either of the biomass crops compared with break crops or cereals, but there was a trend for seedbank densities of the plant groups to be greater in miscanthus than in break or cereal crops. There was no consistent direction of differences in seedbank densities between SRC willows and the arable crops (Fig. 1, Table 2); however, there were lower densities of ruderals in SRC willows than in break crops (*R* = 0.37) and cereals (*R* = 0.38). *Poa annua* L. was the most dominant species in break and cereals crops, while *Matricaria* spp. and *Epilobium* spp. dominated in miscanthus and SRC willows respectively.

**Table 2:** Back‐transformed mean of densities of seeds (counts m^−2^) in the top 0.15 m of soil per field in break crops (break), cereals (cereal), miscanthus (misc) and SRC willows (SRC) and *t*‐statistics for comparisons between biomass and arable crop means, with observed significance levels

	Mean seed density				Comparisons with Miscanthus						Comparisons with SRC					
					Break crops			Cereals			Break crops			Cereals		
	Break	Cereal	Misc	SRC	*t*	*df*	*P*	*t*	*df*	*P*	*t*	*df*	*P*	*t*	*df*	*P*
Total plants	141.2	124.0	206.0	157.9	1.18	249.0	0.240	1.62	97.0	0.109	0.35	249.0	0.728	0.76	97.0	0.450
Monocots	32.5	33.8	61.4	26.7	1.30	249.0	0.196	1.19	97.0	0.239	−0.39	249.0	0.696	−0.45	97.0	0.654
Eudicots	84.7	69.3	129.9	116.8	1.23	249.0	0.221	1.90	97.0	0.060	0.92	249.0	0.359	1.57	97.0	0.120
Ruderals	71.3	68.2	106.6	25.5	0.97	249.0	0.332	1.14	97.0	0.259	−2.46	249.0	0.015	−2.49	97.0	0.014
Competitors	1.7	1.9	1.6	2.5	−0.17	249.0	0.865	−0.45	97.0	0.656	0.72	249.0	0.473	0.38	7.4	0.714

Number of study fields (*N*): break crops = 243; cereals = 91; miscanthus = 8; SRC willows = 8.

**Figure 2:**
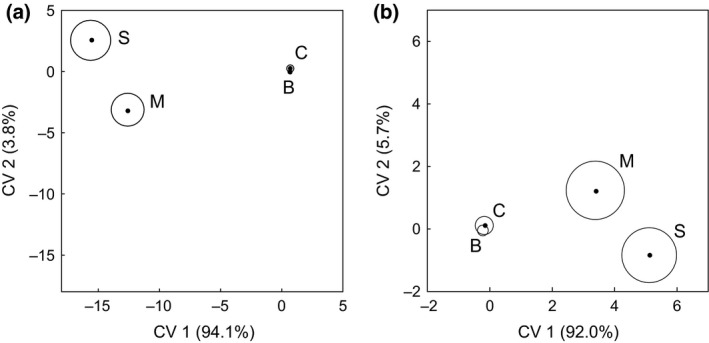
Canonical variates for seedbanks in biomass and arable crops. CVA group mean scores (•) with 95% confidence regions for proportions recorded in the seedbank of (a) taxa and (b) plant growth strategies (after Grime 2001) in break crops (B), cereals (C), miscanthus (M) and SRC willows (S). The percentage variation explained by each canonical variate is given in parentheses.

Canonical variates analysis of the proportion of the densities of 64 taxa recorded from the seedbanks of the four crop types identified distinct communities in miscanthus and SRC willows; however, seedbank communities of cereal and break crops were not distinct from each other (Fig. 2a). The first two axes explained 97.9% of the variation accounting for 94.1% (*χ* ^2^_192_ = 897.97, *P* < 0.001) and 3.8% (*χ* ^2^_126_ = 166.55, *P* < 0.009) of the variation, respectively, for axes 1 and 2.

Canonical variates analysis of the proportion of the densities of ten plant strategies recorded from the seedbanks of the four crop types identified distinct strategy‐based communities in some of the crop types (Fig. 2b). The communities in miscanthus and SRC willows were distinct from each other and from the composite community of break crops and cereals. The first two axes represented 97.7% of the variation, with separation between the biomass crops and the arable crops along axis 1, that accounted for 92.0% (*χ* ^2^_30_ = 246.17, *P* < 0.001) of the variation. Separation along axis 2 was not statistically significant, representing only 5.7% of the variation (*χ* ^2^_18_ = 26.59, *P* < 0.087).

### Weed biomass

**Figure 3:**
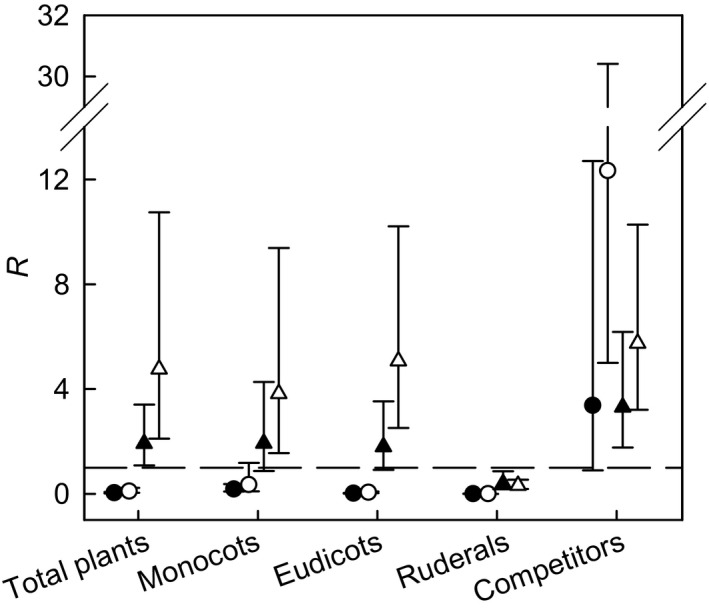
Ratio (*R*) of weed biomass m^−2^ in miscanthus and SRC willows to break and cereal crops. Solid symbols: miscanthus; open symbols: SRC willows; circles: break crops; triangles: cereals. *R* is computed as 10^*d*^, where *d* is the difference between the means (over sites) of the logarithmically transformed densities m^−2^ per field. Dashed line is line of equality (*d* = 0 or *R* = 1). Error bars are 95% confidence limits for *R*, also back‐transformed to the ratio scale (hence asymmetry).

Weed biomass varied between biomass and arable crops, where differences were of many orders of magnitude, ranging from 0.01 to 12.33‐fold (Fig. 3, Table 3). The total amount of weed biomass in both miscanthus and SRC willows was lower than that of break crops (*R* = 0.04; *R* = 0.10, respectively), but higher than that in cereals (*R* = 1.92; *R* = 4.76, respectively). The difference in total weed biomass in miscanthus compared with break crops was reflected in amounts of biomass of monocots (*R* = 0.18), eudicots (*R* = 0.01) and ruderals (*R* = 0.01). There were no differences in amount of competitor biomass in miscanthus compared with break crops. The greater total weed biomass in miscanthus crops compared with cereals was not reflected consistently across the other groupings, with greater competitor biomass (*R* = 3.31) and lower ruderal biomass (*R* = 0.39) in miscanthus. Monocot and eudicot biomasses did not differ between miscanthus and cereals. *Poa annua* L. was the most dominant species in break and cereals crops, while *Cirsium arvense* (L.) and *Elytrigia repens* (L.) dominated miscanthus and SRC willows respectively.

**Table 3:** Back‐transformed mean of weed biomass (g m^−2^) per field in break crops (break), cereals (cereal), miscanthus (misc) and SRC willows (SRC), and *t*‐statistics for comparisons between biomass and arable crop means, with observed significance levels

	Mean biomass				Comparisons with Miscanthus						Comparisons with SRC					
					Break crops			Cereals			Break crops			Cereals		
	Break	Cereal	Misc	SRC	*t*	*df*	*P*	*t*	*df*	*P*	*t*	*df*	*P*	*t*	*df*	*P*
Total plants	613.8	13.3	25.6	63.4	−12.42	21.7	<0.001	2.33	34.0	0.026	−5.50	259.0	<0.001	3.80	97.0	<0.001
Monocots	61.4	6.8	13.2	25.1	−4.82	23.2	<0.001	1.64	100.0	0.104	−1.70	259.0	0.091	2.96	97.0	0.004
Eudicots	404.6	4.6	8.2	23.2	−9.36	262.0	<0.001	1.73	100.0	0.086	−11.02	19.7	<0.001	4.60	97.0	<0.001
Ruderals	150.3	5.2	2.1	1.7	−10.84	262.0	<0.001	−2.35	100.0	0.021	−10.71	259.0	<0.001	−4.43	37.0	<0.001
Competitors	0.4	1.1	3.7	6.5	1.90	21.8	0.070	4.04	17.1	<0.001	5.71	26.7	<0.001	6.45	14.1	<0.001

Number of study fields (*N*): break crops = 247; cereals = 85; miscanthus = 17; SRC willows = 14.

The lower total weed biomass in SRC willows compared with break crops was not reflected across all groupings, where competitor biomass was greater (*R* = 12.33), and biomasses of eudicots and ruderals were lower (*R* = 0.05; *R* = 0.01, respectively). Monocot biomass did not differ between SRC willows and break crops. The greater total weed biomass in SRC willows compared with cereals was reflected in many, but not all, plant groupings. Biomasses of monocot, eudicot and competitor were greater in SRC willows compared with cereals (*R* = 3.82; *R* = 5.07; *R* = 5.75, respectively), but that of ruderal plants was lower (*R* = 0.32).

**Figure 4:**
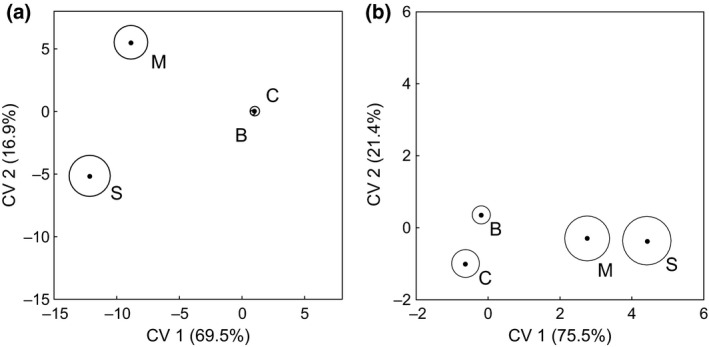
Canonical variates for weed biomass in biomass and arable crops. CVA group mean scores (•) with 95% confidence regions for proportions of biomass of (a) taxa and (b) plant growth strategies (after Grime 2001) in break crops (B), cereals (C), miscanthus (M) and SRC willows (S). The percentage variation explained by each canonical variate is given in parentheses.

Canonical variates analysis of the proportion of biomass of 92 taxa recorded from all four crop types revealed clearly defined species compositions in three of the crop types (Fig. 4a). Just as for seedbanks, communities in miscanthus and SRC willows were distinct both from each other and from those in arable crops, but those in break and cereal crops were indistinguishable. The first two axes accounted for 86.4% of the variation in plant species composition, with clear separation along axis 1 that represented 69.5% of the variation (*χ* ^2^_276_ = 1333.53, *P* < 0.001) and axis 2 that represented 16.85% of the variation (*χ* ^2^_182_ = 653.05, *P* < 0.001).

Canonical variates analysis of the proportion of biomass of nine plant strategies recorded from the four crop types identified distinct strategy‐based communities in all crop types (Fig. 4b). The first two axes explained 96.9% of the variation, accounting for 75.5% (*χ* ^2^_27_ = 357.26, *P* < 0.001) and 21.4% (*χ* ^2^_16_ = 106.88, *P* < 0.001) of the variation, respectively, for axes 1 and 2. The compositions of the plant‐strategy communities in each of the four crop types were distinct from each other, with separation between cereals, miscanthus and SRC willows occurring on axis 1, and separation between the two arable crops types occurring on axis 2.

### Invertebrates

**Figure 5:**
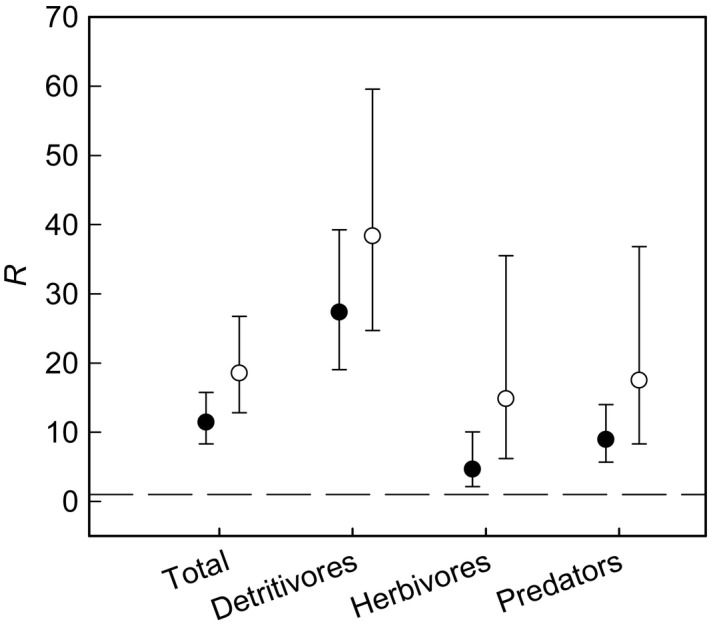
Ratio (*R*) of the density of invertebrates m^−2^ in miscanthus and SRC willows to break crops. Solid symbols: miscanthus; open symbols: SRC willows. *R* is computed as 10^*d*^, where *d* is the difference between the means (over sites) of the logarithmically transformed density of invertebrates m^−2^ per field. Dashed line is line of equality (*d* = 0 or *R* = 1). Error bars are 95% confidence limits for *R*, also back‐transformed to the ratio scale (hence asymmetry).

The densities of all invertebrate groupings were many times greater in the two biomass crops compared with break crops (Fig. 5, Table 4), ranging from 4.64 to 38.37‐fold differences. Isotomid Collembola was the most dominant taxon in both break crops and SRC willows, while entomobryid Collembola were most dominant in miscanthus.

**Table 4:** Back‐transformed mean of densities of invertebrates (counts m^−2^) per field in break crops, miscanthus and SRC willows (SRC), and *t*‐statistics for comparisons between biomass and arable crop means, with observed significance levels

	Mean invertebrate density			Comparisons with Miscanthus			Comparisons with SRC		
	Break crops	Miscanthus	SRC	*t*	*df*	*P*	*t*	*df*	*P*
Total	160.8	1852.5	2998.2	1.18	249.0	0.240	0.35	249.0	0.728
Detritivores	55.6	1547.8	2171.7	1.30	249.0	0.196	−0.39	249.0	0.696
Herbivores	9.7	48.7	157.9	1.23	249.0	0.221	0.92	249.0	0.359
Predators	10.5	101.6	199.9	0.97	249.0	0.332	−2.46	249.0	0.015

Number of study fields (*N*): break crops = 233; miscanthus = 14; SRC willows = 11.

**Figure 6:**
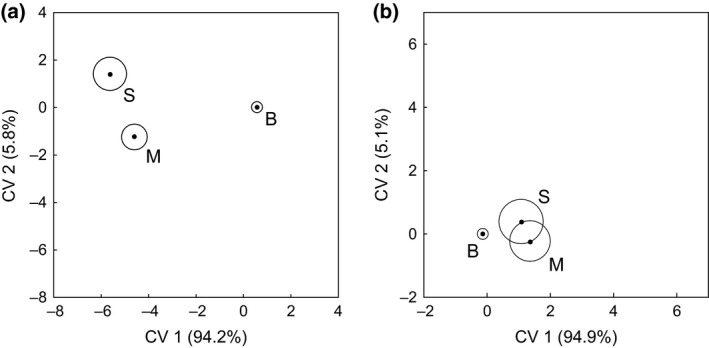
Canonical variates for invertebrates in biomass and break crops. CVA group mean scores (•) with 95% confidence regions for proportions of invertebrate (a) taxa and (b) trophic functional group recorded in break crops (B), miscanthus (M) and SRC willows (S). The percentage variation explained by each canonical variate is given in parentheses.

The defined trophic groups comprised 47% of total invertebrates in break crops, and 92% and 84% in miscanthus and SRC willows, respectively, with detritivores consistently the dominant trophic group, representing 35%, 84% and 72% of total invertebrates in break crops, miscanthus and SRC willows respectively. CVA of the compositions of 41 taxa recorded from the three crop types identified distinct communities (Fig. 6a). The communities were separated on axis 1 (*χ* ^2^_82_ = 357.07, *P* < 0.001) representing 94.2% of the variation, but not on axis 2 (*χ* ^2^_40_ = 38.43, *P* < 0.541). Trophic communities of the two biomass crops were different from those of the break crops, but not from each other (Fig. 6b), with separation along axis 1 (*χ* ^2^_8_ = 43.40, *P* < 0.001) accounting for 94.9% of the variation, but not axis 2 (*χ* ^2^_3_ = 2.39, *P* < 0.495).

## Discussion

Our findings corroborate our, and previous authors' (e.g. (Meehan *et al*., 2013), expectations that replacing annual arable crops with perennial, dedicated biomass crops results in significant, large‐scale changes to the abundance and composition of plant and invertebrate biodiversity indicators. We suggested that such changes would be a result of differences in crop management. Apart from the differences in physical structure of biomass crops, the between‐year timing and frequency of inputs, harvesting and other disturbance in perennial crops are both consistent and reduced in comparison with intensively farmed arable crops that constitute the annual rotation‐based farming system. We believe these characteristics of perennial cropping led to increases in abundance of competitors and decreases in ruderals and we expect these differences to persist through the lifetime of the crop. Similar responses by plant traits to changes in frequency and intensity of crop management have been reported in arable crops (Froud‐Williams *et al*., 1983); field margins (Critchley *et al*., 2006) and set‐aside (Boatman *et al*., 2011). Rowe *et al*. (2011) assessed responses to biomass and cereal crops by plant traits and found a statistically, nonsignificant trend towards greater numbers of perennial plants in biomass crops. It is likely that high variability between the low number of study sites (three) in the study by Rowe *et al*. (2011) contributed to the lack of a statistically significant result and highlights the value of large‐scale studies such as these we report here. Storkey *et al*. (2013) assessed the effects of arable cropping on plant assemblages in greater detail, by measuring the response by plant traits to a disturbance gradient that ranged from annual cultivations and inputs to perennial noncrop habitat and demonstrated that frequency of disturbance was an important driver of trait‐based community assembly in the arable systems tested.

Functional approaches have been argued to provide a more parsimonious explanation and understanding of management effects on biodiversity and ecosystem functioning in comparison to species‐based, taxonomic approaches (Hooper *et al*., 2005; Cadotte *et al*., 2011; Gagic *et al*., 2015). Analysis of functional groups has allowed us greater insight into ecosystem function responses to levels of disturbance, as we note the amount of variation accounted for by the first two axes in the weed CVA was greater for the functional group analyses than in the taxonomic groups (96.9% vs. 86.4% for weed biomass; 97.7% vs. 86.4% for seedbank). Although not measured here, functional indices have been shown to be positively related to ecosystem function (Hoehn *et al*., 2008) and a next step would be to assess the functional diversity of the communities.

We used two contrasting indicators of weed biodiversity: seedbanks and biomass. Seedbanks are a repository of the effect of previous management (Bohan *et al*., 2011), reflecting the longer term effects of field management and cropping system (Hawes *et al*., 2010) and our results appear to be consistent with this, because crop effect ratios (*R*) of seedbank densities in the biomass‐break crops and biomass‐cereal comparisons are similar in terms of magnitude and direction of difference. Measures of weed biomass, however, reflect within‐season effects of growing a particular crop type (Heard *et al*., 2003; Hawes *et al*., 2009), and this has been inferred by Baum *et al*. (2013b) and in our results, as the crop effect ratios (*R*) of weed biomass densities are very different both in their order of magnitude and direction of difference in the biomass‐break crop and biomass‐cereal comparisons. Thus, for a given field, the choice of crop in an annual cropping system results in different effects on abundance of weed taxa and growth strategies between cropping seasons. However, the trend of direction of crop effect on indicators observed in biomass is not seen in the results for seedbanks. Our work suggests that weed biomass could be a predictor of the development of seedbanks in established, perennial crops. Where we recorded significant and consistent crop effects on weed biomass for biomass‐break crops and biomass‐cereal comparisons, we expect the same effects to develop in the seedbank of miscanthus and SRC willows, such that there would be a longer term shift towards a flora dominated by perennials and competitors.

This work has shown responses by biodiversity indicators to crop planting vary according to the type and longevity of the crop. The similarity and consistency in direction and magnitude of crop effect on the seedbank suggest that, when assessed across rotations of an arable system, cereals and break crops are components of a single cropping system. Indeed, the community analyses of weed taxa recorded as biomass and in the seedbank identify a unified arable community. While our findings support the theory of spatially structured arable weed communities (Freckleton & Watkinson, 2002), the regional‐ and national‐scale data used here suggest that these communities operate at greater scales than suggested. Previous analyses of weed and invertebrate communities in arable crops have also identified taxonomic community response to farm management at different temporal and spatial scales. Hawes *et al*. (2010) found longer term, farm‐scale cropping system‐mediated responses by weed seedbank communities in conventional, organic and integrated fields, while within‐year, field‐scale effects of crop were identified by Smith *et al*. (2008), who found that weed and invertebrate communities were associated with individual break crops. Our work demonstrates an intermediate level of response to cropping system, as arable and biomass cropping is possible within a single farm unit.

We have previously reported results for butterfly data collected in this multi‐site, regional‐scale experiment (Haughton *et al*., 2009), where the abundance of nonpest butterfly species was significantly higher, and that of pest butterflies was significantly lower in the field margins of both miscanthus and SRC willows than arable break crops. The results presented here, based on large numbers of entire, commercial fields and study fields distributed regionally and nationally, show greater abundances of biodiversity indicators in biomass crops at the landscape scale. This concurs with the limited number of previous, predominantly small‐scale, local studies of comparative impacts on biodiversity of cultivating miscanthus, SRC willows and arable crops. Bellamy *et al*. (2009) found greater weed cover and abundance of canopy invertebrates in miscanthus than in cereals led to greater numbers and diversity of bird species and Rowe *et al*. (2011) and Baum *et al*. (2012) reported greater weed biomass and species richness, respectively, in SRC willows than in cereals. Stanley & Stout (2013) found significant benefits of miscanthus to solitary, nesting bees compared with cereal crops and suggest this could be a result of the enhanced floral resource in miscanthus. In a similar, but larger, experiment carried out in a different geographical region to Bellamy *et al*. (2009), Sage *et al*. (2010) found fewer bird species and individuals in miscanthus, and suggested that location and differences in the levels of weediness contributed to this disparity in their results, thus indirectly suggesting that biodiversity studies should be widely spatially distributed if they are to account for regional variability. Miscanthus is known to be patchy in its early establishment, which in turn leads to patchy distributions of weeds (Zimmermann *et al*., 2014) and Dauber *et al*. (2015) caution against long‐term expectations of biodiversity benefits of miscanthus, as they suggest farmers would eliminate such patches to maximize crop yield. The fields of miscanthus studied in this experiment were the oldest commercially managed crops available at the time of the experiment and it is possible that they were in the late establishment phase (Karp & Shield, 2008). Nevertheless, the study fields were managed for yield, and we would expect patches of weeds in these late establishment phase crops to remain a feature in older crops.

An unexpected and surprising outcome of this work was the contrast in the magnitudes of the crop effect on weed biomass and the invertebrates, suggesting that, unlike in arable crops, there is a significant positive crop–resource relationship in perennial biomass crops. Previous studies in annual arable crops (Hawes *et al*., 2003, 2009; Bohan *et al*., 2007) have demonstrated positive relationships between weed resource and invertebrate functional groups, and we suggest that in addition to the effect of the biomass crop itself, the competitor plant community in biomass crops could, in part, drive the detritivore‐dominated invertebrate fauna in biomass crops, as the competitors were the only group of plants to show greater densities in biomass crops than break crops. Competitor plant types typically exhibit a perennial reproductive strategy (Grime *et al*., 2007) and positive benefits of perennial vegetation on species richness and abundance of parasitic tachinid Diptera have been reported by Letourneau *et al*. (2012). Storkey *et al*. (2013) found that plant herbivores respond positively to ruderal plants in arable systems; however, we found no evidence of this in perennial biomass crops, as the density of plant herbivores was greater in biomass crops, despite statistically significant lower densities of ruderal plant biomass. It is unfortunate that similar data for invertebrates in cereals were not collected in the FSEs, and to our knowledge, are not available elsewhere at scales equivalent to those analysed here; however, if the same pattern we have found in break crops were followed in cereals, we would predict that invertebrate abundance could be somewhat greater in cereals, due to the marginally greater densities of competitors than in break crops.

In conclusion, our analyses of regional‐ and national‐scale data have shown that indicators of biodiversity are more abundant in perennial biomass cropping systems than annual cropping systems and we identified divergent functional compositions of plant and invertebrate communities in the arable and biomass crops. Our analyses also confirm the value of break crops for biodiversity indicators in arable rotations. These findings support the view that strategic planting of dedicated biomass crops, in intensively managed, arable‐dominated farmland, can be used as a powerful tool for increasing landscape heterogeneity in the bid to create resilient, multifunctional landscapes (Rader *et al*., 2014).

## Supporting information


**Table S1.** Biomass crop study site details.

**Table S2.** Allocation of plant species to phylogenetic and growth strategy groups.

## References

[gcbb12312-bib-0001] APG (2003) An update of the Angiosperm Phylogeny Group classification for the orders and families of flowering plants: APG II. Botanical Journal of the Linnean Society, 141, 399–436.

[gcbb12312-bib-0501] Arnold AJ (1994) Insect suction sampling without nets, bags or filters. Crop Protection, 13, 73–76.

[gcbb12312-bib-0002] Baum S , Bolte A , Weih M (2012) High value of short rotation coppice plantations for phytodiversity in rural landscapes. Global Change Biology Bioenergy, 4, 728–738.

[gcbb12312-bib-0003] Baum C , Eckhardt KU , Hahn J , Weih M , Dimitriou I , Leinweber P (2013a) Impact of poplar on soil organic matter quality and microbial communities in arable soils. Plant Soil and Environment, 59, 95–100.

[gcbb12312-bib-0004] Baum S , Weih M , Bolte A (2013b) Floristic diversity in Short Rotation Coppice (SRC) plantations: comparison between soil seed bank and recent vegetation. Landbauforschung, 63, 221–228.

[gcbb12312-bib-0005] Bellamy PE , Croxton PJ , Heard MS *et al* (2009) The impact of growing miscanthus for biomass on farmland bird populations. Biomass and Bioenergy, 33, 191–199.

[gcbb12312-bib-0006] Benton TG , Vickery JA , Wilson JD (2003) Farmland biodiversity: is habitat heterogeneity the key? Trends in Ecology & Evolution, 18, 182–188.

[gcbb12312-bib-0007] Berndes G , Borjesson P , Ostwald M , Palm M (2008) Multifunctional biomass production systems ‐ an overview with presentation of specific applications in India and Sweden. Biofuels Bioproducts & Biorefining, 2, 16–25.

[gcbb12312-bib-0008] Bianchi F , Booij CJH , Tscharntke T (2006) Sustainable pest regulation in agricultural landscapes: a review on landscape composition, biodiversity and natural pest control. Proceedings of the Royal Society B‐Biological Sciences, 273, 1715–1727. 10.1098/rspb.2006.3530 PMC1634792 16790403

[gcbb12312-bib-0009] Boatman ND , Jones NE , Conyers ST , Pietravalle S (2011) Development of plant communities on set‐aside in England. Agriculture, Ecosystems and Environment, 143, 8–19.

[gcbb12312-bib-0010] Bohan DA , Boffey CWH , Brooks DR *et al* (2005) Effects on weed and invertebrate abundance and diversity of herbicide management in genetically modified herbicide‐tolerant winter‐sown oilseed rape. Proceedings of the Royal Society B, 272, 463–474. 15799941 10.1098/rspb.2004.3049 PMC1578713

[gcbb12312-bib-0011] Bohan DA , Hawes C , Haughton AJ , Denholm I , Champion GT , Perry JN , Clark SJ (2007) Statistical models to evaluate invertebrate‐plant trophic interactions in arable systems. Bulletin of Entomological Research, 97, 265–280. 17524158 10.1017/S0007485307004890

[gcbb12312-bib-0012] Bohan DA , Powers SJ , Champion G *et al* (2011) Modelling rotations: can crop sequence explain arable weed seedbank abundance? Weed Research, 51, 422–432.

[gcbb12312-bib-0013] Bourke D , Stanley D , O'rourke E *et al* (2014) Response of farmland biodiversity to the introduction of bioenergy crops: effects of local factors and surrounding landscape context. Global Change Biology Bioenergy, 6, 275–289.

[gcbb12312-bib-0014] Cadotte MW , Carscadden K , Mirotchnick N (2011) Beyond species: functional diversity and the maintenance of ecological processes and services. Journal of Applied Ecology, 48, 1079–1087.

[gcbb12312-bib-0015] Champion GT , May MJ , Bennett S *et al* (2003) Crop management and agronomic context of the Farm Scale Evaluations of genetically modified herbicide‐tolerant crops. Philosophical Transactions of the Royal Society, 358, 1801–1818. 10.1098/rstb.2003.1405 PMC1693273 14561315

[gcbb12312-bib-0016] Coghlan A (2003) Farming 1, wildlife 0. New Scientist, 2418, 21.

[gcbb12312-bib-0017] Critchley CNR , Fowbert JA , Sherwood AJ (2006) The effects of annual cultivation on plant community composition of uncropped arable field boundary strips. Agriculture, Ecosystems and Environment, 113, 196–205.

[gcbb12312-bib-0018] Dauber J , Bolte A (2014) Bioenergy: challenge or support for the conservation of biodiversity? Global Change Biology Bioenergy, 6, 180–182.

[gcbb12312-bib-0019] Dauber J , Jones MB , Stout JC (2010) The impact of biomass crop cultivation on temperate biodiversity. Global Change Biology Bioenergy, 2, 289–309.

[gcbb12312-bib-0020] Dauber J , Cass S , Gabriel D , Harte K , Astrom S , O'rourke E , Stout JC (2015) Yield‐biodiversity trade‐off in patchy fields of *Miscanthus x giganteus* . Global Change Biology Bioenergy, 7, 455–467.

[gcbb12312-bib-0021] Davis SJ , Shearer C (2014) A crack in the natural‐gas bridge. Nature, 514, 436–437. 25317563 10.1038/nature13927

[gcbb12312-bib-0022] Defra (2014) UK Biodiversity Indicators 2014. Defra, London, UK.

[gcbb12312-bib-0023] Donald PF , Green RE , Heath MF (2001) Agricultural intensification and the collapse of Europe's farmland bird populations. Proceedings of the Royal Society B, 268, 25–29. 12123294 10.1098/rspb.2000.1325 PMC1087596

[gcbb12312-bib-0024] Fargione J (2010) Is bioenergy for the birds? An evaluation of alternative future bioenergy landscapes. Proceedings of the National Academy of Sciences of the United States of America, 107, 18745–18746. 20962276 10.1073/pnas.1014045107 PMC2973879

[gcbb12312-bib-0025] Firbank LG , Heard MS , Woiwod IP *et al* (2003) An introduction to the Farm‐Scale Evaluations of genetically modified herbicide‐tolerant crops. Journal of Applied Ecology, 40, 2–16.

[gcbb12312-bib-0026] Freckleton RP , Watkinson AR (2002) Large‐scale spatial dynamics of plants: metapopulations, regional ensembles and patchy populations. Journal of Ecology, 90, 419–434.

[gcbb12312-bib-0027] Froud‐Williams RJ , Chancellor RJ , Drennan DSH (1983) Influence of cultivation regime on weed floras of arable cropping systems. Journal of Applied Ecology, 20, 187–197.

[gcbb12312-bib-0028] Gagic V , Bartomeus I , Jonsson T *et al* (2015) Functional identity and diversity of animals predict ecosystem functioning better than species‐based indices. Proceedings of the Royal Society B, 282, 20142620. 25567651 10.1098/rspb.2014.2620 PMC4309003

[gcbb12312-bib-0029] Gardner S , Gower JC , Le Roux NJ (2006) A synthesis of canonical variate analysis, generalised canonical correlation and Procrustes analysis. Computational Statistics & Data Analysis, 50, 107–134.

[gcbb12312-bib-0504] Grime JP (2001) Plant Strategies, Vegetation Processes, and Ecosystem Processes, 2nd edn John Wiley and Sons, Chichester.

[gcbb12312-bib-0030] Grime JP , Hodgson JC , Hunt R (2007) Comparative Plant Ecology: A Functional Approach to Common British Species. Castlepoint, Dalbeattie.

[gcbb12312-bib-0031] Haughton AJ , Champion GT , Hawes C *et al* (2003) Invertebrate responses to the management of genetically modified herbicide‐tolerant and conventional spring crops. II. Within‐field epigeal and aerial arthropods. Philosophical Transactions of the Royal Society, 358, 1863–1877. 10.1098/rstb.2003.1408 PMC1693277 14561319

[gcbb12312-bib-0032] Haughton AJ , Bond AJ , Lovett AA *et al* (2009) A novel, integrated approach to assesseing social, economic and environmental implications of changing rural land‐use: a case study of perennial biomass crops. Journal of Applied Ecology, 46, 315–322.

[gcbb12312-bib-0033] Hawes C , Haughton AJ , Osborne JL *et al* (2003) Responses of plants and invertebrate trophic groups to contrasting herbicide regimes in the Farm Scale Evaluations of genetically modified herbicide‐tolerant corops. Philosophical Transactions of the Royal Society, 358, 1899–1913. 10.1098/rstb.2003.1406 PMC1693274 14561321

[gcbb12312-bib-0034] Hawes C , Haughton AJ , Bohan DA , Squire GR (2009) Functional approaches for assessing plant and invertebrate abundance patterns in arable systems. Basic and Applied Ecology, 10, 34–42.

[gcbb12312-bib-0035] Hawes C , Squire GR , Hallett PD , Watson CA , Young M (2010) Arable plant communities as indicators of farming practice. Agriculture, Ecosystems and Environment, 138, 17–26.

[gcbb12312-bib-0036] Heard MS , Hawes C , Champion GT *et al* (2003) Weeds in fields with contrasting conventional and genetically modified herbicide‐tolerant crops. I. Effects on abundance and diversity. Philosophical Transactions of the Royal Society, 358, 1818–1822. 10.1098/rstb.2003.1402 PMC1693279 14561316

[gcbb12312-bib-0037] Heard MS , Rothery P , Perry JN , Firbank LG (2005) Predicting longer‐term changes in weed populations under GMHT crop management. Weed Research, 45, 331–338.

[gcbb12312-bib-0038] Hoehn P , Tscharntke T , Tylianakis JM , Steffan‐Dewenter I (2008) Functional group diversity of bee pollinators increases crop yield. Proceedings of the Royal Society B, 275, 2283–2291. 18595841 10.1098/rspb.2008.0405 PMC2603237

[gcbb12312-bib-0502] Holland RA , Eigenbrod F , Muggeridge A , Brown G , Clarke D , Taylor G (2015) A synthesis of the ecosystem services impact of second generation bioenergy crop production. Renewable and Sustainable Energy Reviews, 46, 30–40.

[gcbb12312-bib-0039] Hooper DU , Chapin FS , Ewel JJ *et al* (2005) Effects of biodiversity on ecosystem functioning: a consensus of current knowledge. Ecological Monographs, 75, 3–35.

[gcbb12312-bib-0040] IPCC (2014) Climate Change 2014: Synthesis Report. Contribution of Working Groups I, II and III to the Fifth Assessment Report of the Intergovernmental Panel on Climate Change (eds Core Writing Team , Pachauri RK, Meyer LA). IPCC, Geneva, Switzerland.

[gcbb12312-bib-0041] Jackson RB , Vengosh A , Carey JW , Davies RJ , Darrah TH , O'sullivan F , Petron G (2014) The environment costs and benefits of fracking. Annual Reviews of Environment and Resources, 39, 327–362.

[gcbb12312-bib-0042] Karp A , Shield I (2008) Bioenergy from plants and the sustainable yield challenge. New Phytologist, 179, 15–32. 18422906 10.1111/j.1469-8137.2008.02432.x

[gcbb12312-bib-0503] Karp A , Haughton AJ , Bohan DA et al. (2009) Perennial energy crops: implications and potential In: What is Land For? The Food, Fuel and Climate Change Debate (eds Winter M, Lobley M), pp. 47–72. Earthscan, London.

[gcbb12312-bib-0043] Letourneau DK , Bothwell Allen SG , Stireman JO (2012) Perennial habitat fragments, parasitoid diversity and parasitism in ephemeral crops. Journal of Applied Ecology, 49, 1405–1416.

[gcbb12312-bib-0044] McJeon H , Edmonds J , Bauer N *et al* (2014) Limited impact on decadal‐scale climate change from increased use of natural gas. Nature, 514, 482–485. 25317557 10.1038/nature13837

[gcbb12312-bib-0045] Meehan TD , Hurlbert AH , Gratton C (2010) Bird communities in future bioenergy landscapes of the Upper Midwest. Proceedings of the National Academy of Sciences of the United States of America, 107, 18533–18538. 20921398 10.1073/pnas.1008475107 PMC2972996

[gcbb12312-bib-0046] Meehan TD , Gratton C , Diehl E *et al* (2013) Ecosystem‐service tradeoffs associated with switching from annual to perennial energy crops in riparian zones of the US Midwest. PLoS ONE, 8. doi: 10.1371/journal.pone.0080093. 10.1371/journal.pone.0080093 PMC3819318 24223215

[gcbb12312-bib-0047] Perry JN , Rothery P , Clark SJ , Heard MS , Hawes C (2003) Design, analysis and statistical power of the Farm‐Scale Evaluations of genetically modified herbicide‐tolerant crops. Journal of Applied Ecology, 40, 17–31.

[gcbb12312-bib-0048] Rader R , Birkhofer K , Schmucki R , Smith HG , Stjernman M , Lindborg R (2014) Organic farming and heterogeneous landscapes positively affect different measures of plant diversity. Journal of Applied Ecology, 51, 1544–1553.

[gcbb12312-bib-0049] Rowe RL , Goulson D , Street NR , Taylor G (2009) Identifying potential environmental impacts of large‐scale deployment of dedicated bioenergy crops in the UK. Renewable & Sustainable Energy Reviews, 13, 271–290.

[gcbb12312-bib-0050] Rowe RL , Hanley ME , Goulson D , Clarke DJ , Doncaster CP , Taylor G (2011) Potential benefits of commercial willow Short Rotation Coppice (SRC) for farm‐scale plant and invertebrate communities in the agri‐environment. Biomass and Bioenergy, 35, 325–336.

[gcbb12312-bib-0051] Sage R , Cunningham M , Haughton AJ , Mallott MD , Bohan DA , Riche A , Karp A (2010) The environmental impacts of biomass crops: use by birds of miscanthus in summer and winter in southwestern England. IBIS, 152, 487–499.

[gcbb12312-bib-0052] Searchinger T , Edwards R , Mulligan D , Heimlich R , Plevin R (2015) Do biofuel policies seek to cut emissions by cutting food? Science, 347, 1420–1422. 25814570 10.1126/science.1261221

[gcbb12312-bib-0053] Senapathi D , Carvalheiro LG , Biesmeijer JC *et al* (2015) The impact of over 80 years of land cover changes on bee and wasp pollinator communities in England. Proceedings of the Royal Society B, 282. doi: 10.1098/rspb.2015.0294. 10.1098/rspb.2015.0294 PMC4426632 25833861

[gcbb12312-bib-0054] Smith V , Bohan DA , Clark SJ , Haughton AJ , Bell JR , Heard MS (2008) Weed and invertebrate community compositions in arable farmland. Arthropod‐Plant Interactions, 2, 21–30.

[gcbb12312-bib-0055] Sokal RR , Rohlf FJ (2012) Biometry: The Principles and Practice of Statistics in Biological Research. Freeman and Co, New York.

[gcbb12312-bib-0056] Stace C (2010) New Flora of the British Isles. Cambridge University Press, Cambridge.

[gcbb12312-bib-0057] Stanley DA , Stout JC (2013) Quantifying the impacts of bioenergy crops on pollinating insect abundance and diversity: a field‐scale evaluation reveals taxon‐specific responses. Journal of Applied Ecology, 50, 335–344.

[gcbb12312-bib-0058] Storkey J , Meyer S , Still KS , Leuschner C (2012) The impact of agricultural intensification and land‐use change on the European arable flora. Proceedings of the Royal Society B, 279, 1421–1429. 21993499 10.1098/rspb.2011.1686 PMC3282365

[gcbb12312-bib-0059] Storkey J , Brooks D , Haughton A , Hawes C , Smith BM , Holland JM (2013) Using functional traits to quantify the value of plant communities to invertebrate ecosystem service providers in arable landscapes. Journal of Ecology, 101, 38–46.

[gcbb12312-bib-0060] Tilman D , Socolow R , Foley JA *et al* (2009) Beneficial biofuels‐the food, energy, and environment trilemma. Science, 325, 270–271. 19608900 10.1126/science.1177970

[gcbb12312-bib-0061] VSNI (2014) GenStat for Windows, 17th edn VSN International, Hemel Hempstead.

[gcbb12312-bib-0062] Woodcock BA , Harrower C , Redhead J *et al* (2014) National patterns of functional diversity and redundancy in predatory ground beetles and bees associated with key UK arable crops. Journal of Applied Ecology, 51, 142–151.

[gcbb12312-bib-0063] Zimmermann J , Styles D , Hastings A , Dauber J , Jones MB (2014) Assessing the impact of within crop heterogeneity (‘patchiness’) in young *Miscanthus × giganteus* fields on economic feasibility and soil carbon sequestration. Global Change Biology Bioenergy, 6, 566–576.

